# New Criteria for Lupus

**DOI:** 10.1007/s11926-020-00896-6

**Published:** 2020-05-13

**Authors:** Martin Aringer, Nicolai Leuchten, Sindhu R. Johnson

**Affiliations:** 1 Division of Rheumatology, Department of Medicine III, University Medical Center and Faculty of Medicine Carl Gustav Carus at the TU Dresden, Fetscherstrasse 74, 01307 Dresden, Germany; 2 Division of Rheumatology, Department of Medicine, Toronto Western Hospital, Mount Sinai Hospital; Institute of Health Policy, Management and Evaluation, University of Toronto, Toronto, Ontario Canada; 3 Toronto Western Hospital, 399 Bathurst Street, Toronto, Ontario M5T 2S8 Canada

**Keywords:** Systemic lupus erythematosus, Classification criteria, Antinuclear antibodies, Cutaneous lupus erythematosus, Arthritis, Nephritis

## Abstract

**Purpose of the Review:**

Classification criteria define the patient population for clinical trials and translational studies, but also influence current understanding of the disease. This review attempts to delineate the development from the American College of Rheumatology (ACR) 1982 to the European League Against Rheumatism (EULAR)/ACR 2019 classification criteria for systemic lupus erythematosus (SLE).

**Recent Findings:**

The new EULAR/ACR classification criteria use antinuclear antibodies (ANA) as an entry criterion. (Non-infectious) fever is the one new criterion. All criteria items now have individual weights (from 2 to 10) and are structured in domains, within which only the highest item is counted. There is one common attribution rule, counting criteria only if there is no more likely alternative explanation. Ten points are sufficient for classification. The new criteria have reached a sensitivity of 96.1% and a specificity of 93.4%.

**Summary:**

The new EULAR/ACR 2019 classification criteria for SLE build on the previous criteria sets, adding fever only as a new criteria item. The new structure is reflective of the current diagnostic approach and has led to improved statistical performance.

## Introduction

In several ways, systemic lupus erythematosus (SLE) is a rather unusual disease. Essentially, all organ systems can get involved [1–3]. For many of the organs, involvement can manifest in more than one way. On the other hand, no symptom is given and the disease of two SLE patients can differ in every possible way. Diagnosing SLE therefore poses challenges [4], and teaching lupus is not a trivial task. With its even higher demand for specificity at the time of inclusion into clinical trials and translational studies, classification is at the same time challenging and key.

The 1982 American College of Rheumatology (ACR) classification criteria for SLE [5] constituted a ground-breaking effort that shaped SLE science. These criteria subsequently influenced teaching and, more indirectly, clinical diagnosis over decades. Their 1997 revision mainly added the anti-phospholipid antibodies [6], reflecting a major advance in the field. For their time, classification based on the presence of four out of 11 individual criteria was optimal. These criteria need no introduction and almost all physicians have learned them by heart. Since, content-wise, additions have been relatively limited over time, we will use the single ACR criteria items to go over the changes implemented for the 2012 Systemic Lupus International Collaborating Clinics (SLICC) criteria [7] and the new 2019 European League Against Rheumatism (EULAR)/ACR classification criteria [8•, 9•], discussing the rationale on the way.

One of the key elements that were changed for the EULAR/ACR criteria in comparison to the older sets of criteria is attribution. Both the 1982/1997 ACR classification criteria and the 2012 SLICC criteria defined exclusion criteria for various items. For one example, thrombocytopenia was defined by the ACR criteria as less than 100,000/mm^3^ in the absence of offending drugs [5], by the SLICC criteria as < 100,000/mm^3^ at least once in the absence of other known causes such as drugs, portal hypertension, and thrombotic thrombocytopenic purpura [7]. Since this list was already of considerable length for the SLICC criteria, but would have become even much longer if even remotely complete, the EULAR/ACR criteria define only one attribution rule for all items, namely that items are only to be counted towards SLE if there is no more likely other explanation [8•, 9•, 10, 11]. This is in line with expert diagnosis and also allows for patients with SLE overlap syndromes, such as Rhupus (rheumatoid arthritis plus SLE) [12, 13], to be classified as SLE patients. This essential feature is key to correctly employing the new criteria [14], and thus like an overarching principle needed to be mentioned before going into the various organ systems.

Another key feature that needs to be discussed beforehand is the weighting in the EULAR/ACR criteria. To some degree, the SLICC criteria already introduced different weights in that lupus nephritis by histology plus antibodies was found sufficient for classification [7], which usually needed 4 points. Thus, histological lupus nephritis had an effective weight of 3 points (adding to 1 for the antibodies). In expert diagnosis, it is evident that various items are given grossly different weights. With the possibility to now scientifically evaluate such weights by multicriteria decision analysis [15], it appeared logical to take this approach, an essential part of the new EULAR/ACR criteria. In the end, 10 points are sufficient for classification, and weights start at 2 (e.g. for oral ulcers) and end at 10 (class III or IV lupus nephritis). With these two novelties introduced, we will now continue with the organ systems and the changes of the corresponding criteria.

## Malar Rash

The prototypical butterfly-shaped skin lesion of SLE, which has also led many of the patient organizations to identify with the butterfly, has in essence retained its position over time (Table 1). Defined as “fixed erythema, flat or raised, over the malar eminences, tending to spare the nasolabial folds” [5], it sometimes poses problems in differential diagnosis, mainly against rosacea and occasionally against dermatomyositis [16]. If this skin symptom is correctly diagnosed, however, SLE is rather likely. Malar rash is the most common form of what now is defined as acute cutaneous lupus erythematosus (ACLE), and the SLICC criteria have accordingly transformed the item to ACLE, including malar rash, bullous lupus, the toxic epidermal necrolysis variant of SLE, maculopapular lupus rash, and photosensitive lupus rash (in the absence of dermatomyositis) [7]. For the EULAR/ACR criteria, this list was reduced to malar rash or a generalized maculopapular rash observed by a clinician, allowing for photographs being read. This was based on the very low prevalence of the other forms and the necessity to be brief for allowing the criteria to be feasible. ACLE in this definition is a relatively heavy item in the EULAR/ACR criteria, reaching 6 of the 10 points necessary for classification.

**Table 1:** Single criteria in the four SLE classification criteria sets since 1982

ACR 1982	ACR 1997	SLICC 2012	EULAR/ACR 2019	
			Mucocutaneous	
1. Malar rash		1. Acute cutaneous LE*	Acute cutaneous LE	6
		or SCLE	SCLE	4
2. Discoid rash		2. Chronic cutaneous LE*	Discoid LE	4
3. Photosensitivity				
4. Oral ulcers		3. Oral ulcers	Oral ulcers	2
		or nasal ulcers		
		4. Non-scarring alopecia	Non-scarring alopecia	2
5. Arthritis		5. Synovitis	Joint involvement	6
6. Serositis		6. Serositis	Serosal	
a) Pleuritis		Pleuritis	Effusion	5
b) Pericarditis		or pericarditis	Acute pericarditis	6
7. Renal disorder		7. Renal	Renal	
a) Persistent proteinuria		Proteinuria	Proteinuria	4
b) Cellular casts		or red cell casts		
		Histology compatible with lupus nephritis	ISN/RPS II/V	8
			ISN/RPS III/IV	10
8.Neurologic disorder		8. Neurologic	Neuropsychiatric	
a) Seizures		Seizures	Seizure	5
b) Psychosis		Psychosis	Psychosis	3
		Mononeuritis multiplex		
		Myelitis		
		Peripheral or cranial neuropathy		
		Acute confusional state	Delirium	2
9. Hematologic disorder			Hematologic	
a) Hemolytic anemia		9. Hemolytic anemia	Coombs+ hemolytic anemia	4
b) Leukopenia		10. Leukopenia	Leukopenia	3
c) Lymphopenia		or lymphopenia		
d) Thrombocytopenia		11. Thrombocytopenia	Thrombocytopenia	4
10. Immunologic disorder				
a) LE cell preparation				
			SLE-specific antibodies	
b) Anti-DNA	a) Anti-DNA	12. Anti-dsDNA	Anti-dsDNA	6
c) Anti-Sm	b) Anti-Sm	13. Anti-Sm	Anti-Sm	6
d) False-positive syphilis serology	c) Anti-phospholipid	14. Anti-phospholipid	Anti-phospholipid	2
		15. Low complements	Low complement	
			C3 or C4 low	3
			C3 and C4 low	4
		16. Coombs test without hemolytic anemia		
11. ANA	11. ANA	17. ANA	Entry criterion ANA	

## New Since SLICC Criteria: SCLE

To the ACLE criteria item, the SLICC criteria also added a newer entity, namely subacute cutaneous lupus erythematosus (SCLE) [7, 17]. Not yet present in the ACR criteria, SCLE certainly is a relatively specific item [16]. The EULAR/ACR criteria therefore kept SCLE, but made it an item of its own, specifying the diagnosis as “annular or papulosquamous (psoriasiform) cutaneous eruption, usually photodistributed” [10], which must be observed by a clinician, but with photographs acceptable. Following this differentiation, the multiparameter decision analysis also ended with a lower weight, namely 4 in the final version, attributed to SCLE [8•, 9•].

## Discoid Rash

Although scarring discoid lesions of discoid lupus erythematosus (DLE) more often occur as an isolated skin disease (cutaneous lupus erythematosus) than ACLE and SCLE, discoid rash still is a very typical lupus lesion. DLE is the most common manifestation of chronic cutaneous lupus erythematosus (CCLE). In the SLICC criteria, the CCLE item differentiated between localized and generalized DLE, but also includes a variety of other lesions, namely hypertrophic (verrucous) lupus, lupus panniculitis (lupus profundus), mucosal lupus, lupus erythematosus tumidus, Chilblains lupus, and discoid lupus/lichen planus overlap [7]. The opinion was split over this list. While some valued the completeness of skin lupus symptoms, others found the list much too long. Indeed, most of these items have a very low prevalence in SLE. For the EULAR/ACR criteria, CCLE therefore again became discoid lupus, and the distinction between localized and generalized DLE was omitted based on a lack of consequences. Discoid rash, like SCLE, has a weight of 4 points in the new criteria [8•, 9•].

## Photosensitivity

Defined in the ACR criteria as “skin rash as a result of unusual reaction to sunlight, by patient history or physician observation” [5], photosensitivity certainly is a common symptom in SLE. However, there are issues with sensitivity. Polymorphic light eruption is much more common than SLE, and redness occurring early after UV exposure is easier to remember for patients than cutaneous lupus manifestations, which develop several days to a week after UV exposure. Other issues in differential diagnosis occur in rosacea, which likewise responds to sunlight, and in dermatomyositis [16]. In addition, importantly, photosensitivity cannot be clearly distinguished from cutaneous lupus manifestations. Accordingly, this item lost its independence in the SLICC criteria, ending with ACLE, as listed above. This proved accurate when the interdependence of items was investigated for the EULAR/ACR criteria, demonstrating a clear association of photosensitivity with malar rash. In the EULAR/ACR criteria, photosensitivity still is recalled in the photodistribution of SCLE and DLE [8•, 9•], but has otherwise disappeared.

## Oral Ulcers

Like the other mucocutaneous manifestations, oral ulcers, which occurred in a quarter of the SLE patients in the ACR criteria cohort [5], were scrutinized in the development of both the SLICC and the EULAR/ACR criteria. In contrast to photosensitivity, however, they stood the test of time. The SLICC criteria added nasal ulcers into the item and kept mucosal ulcers independent, with a sensitivity of 44% and a specificity of 92% in the derivation cohort [7]. In the EULAR/ACR criteria, the prevalence of isolated nasal ulcers was found too low, reverting to oral ulcers with a weight of 2 for limited specificity.

## New since SLICC Criteria: Non-scarring Alopecia

While not part of the ACR criteria, hair loss is a fairly common symptom SLE patients complain about. While scarring alopecia in SLE is usually a consequence of a discoid lupus rash (above), the non-scarring variant is even more prevalent. The SLICC group added non-scarring alopecia, defined as diffuse thinning or hair fragility with visible broken hairs to their criteria, with a sensitivity of 32% and a specificity of 96% in their derivation cohort [7]. Non-scarring alopecia observed by a clinician was retained in the EULAR/ACR criteria but with a relatively low weight of 2 points.

## Why a Mucocutaneous Domain

While each of the five remaining mucocutaneous items has their impact, they are not entirely independent. The analysis performed within the EULAR/ACR criteria project clearly showed associations [18]. This is also in line with concepts that skin and mucosal associations and skin rashes and alopecia may well be related. Also, the combination of two of the high-scoring mucocutaneous items in some instances would be sufficient for classification, which could confer risks. Therefore, the EULAR/ACR classification criteria now have one mucocutaneous domain, within which only the highest scoring item is being scored for classification [8•, 9•, 11]. They still retained excellent sensitivity.

## Arthritis

Non-erosive arthritis of at least two joints is a classical SLE feature, with a sensitivity of 79%, but a specificity of only 44% in the SLICC criteria cohort. Arthritis is also one manifestation that necessitates therapy, given both pain and inflammation and the risk of Jaccoud-like damage. The SLICC group introduced the definition of “either synovitis involving 2 or more joints characterized by swelling or effusion or tenderness in 2 or more joints and at least 30 minutes of morning stiffness” [7]. This definition was tested against palpable synovitis in the EULAR/ACR classification criteria project and proved superior. This is also in line with findings that sonography sees inflammatory joint involvement in many patients without frank synovitis. In consequence, arthritis as a term was changed to “joint involvement” in the EULAR/ACR criteria, scoring 6 points [8•, 9•]. Other musculoskeletal symptoms, such as myositis, were too uncommon to be included and also lack specificity.

## Serositis

Like skin and joint manifestations, pleuritis and pericarditis belong to the typical spectrum of SLE organ involvement with 35% sensitivity and 97% specificity in the SLICC derivation cohort [7]. Defined as “pleuritis - convincing history of pleuritic pain or rub heard by a physician or evidence of pleural effusion or pericarditis - documented by ECG or rub or evidence of pericardial effusion” in the ACR criteria [5] and as “typical pleurisy for more than 1 day or pleural effusions or pleural rub” for pleuritis and “typical pericardial pain (pain with recumbency improved by sitting forward) for more than 1 day or pericardial effusion or pericardial rub or pericarditis by electrocardiography” for pericarditis in the SLICC criteria [7], and in the “absence of other causes, such as infection, uremia, and Dressler’s pericarditis”. In the EULAR/ACR criteria process, this item was provisionally split into pleural or pericardial effusion, defined as “imaging evidence (such as ultrasound, x-ray, CT scan, MRI) of pleural or pericardial effusion, or both” and acute pericarditis. For the later, the Cardiology definition of “at least 2 of (i) pericardial chest pain (typically sharp, worse with inspiration, improved by leaning forward), (ii) pericardial rub, (iii) EKG with new widespread ST-elevation or PR depression, (iv) new or worsened pericardial effusion on imaging (such as ultrasound, x-ray, CT scan, MRI)” was adopted. The distinction between pleural or pericardial effusion and acute pericarditis was maintained after the multiparameter decision analysis ended in (slightly) different weights for the two items, namely 5 points for pleural or pericardial effusion and 6 points for acute pericarditis.

## Renal Disorder

Lupus nephritis as the most common dangerous organ manifestation of SLE over time underwent significant changes in definition and weighting. In the ACR 1982 classification criteria, renal disorder was defined as “persistent proteinuria greater than 0.5 grams per day or greater than 3+ if quantitation not performed or cellular casts that may be red cell, hemoglobin, granular, tubular, or mixed” [5] and counted on par with all other criteria items. Since then, renal biopsy had become the standard for diagnosing lupus nephritis, while the protein/creatinine ratio often replaced 24 h proteinuria. Both developments were represented in the SLICC 2012 criteria, which counted a “renal urine protein-to-creatinine ratio (or 24-hour urine protein) representing 500 mg protein/24 hours or red blood cell casts” as a standard item, but also defined that biopsy-proven nephritis compatible with SLE in the presence of ANAs or anti-dsDNA antibodies was sufficient for classifying SLE, thus giving renal histology three times the weight of the other items [7]. While the introduction of lupus nephritis by histology into the SLICC criteria was warmly welcomed worldwide, the EULAR/ACR project refined this approach. In a first step, the decision was made to classify lupus nephritis by the International Society of Nephrology (ISN)/ Renal Pathology Society (RPS) classification. Subsequently, it became obvious that the involved lupus experts saw a difference between severe proliferative lupus nephritis, i.e., ISN/RPS classes III and IV on the one hand and between membranous lupus nephritis (class V) and milder mesangial proliferative lupus nephritis (class II) on the other. While class III and IV nephritis were assigned 10 points, sufficient for classification, class II or V nephritis reached a slightly lower weight of 8 points and therefore need another criteria item for classification. This is due to a somewhat lesser specificity of membranous or mesangial proliferative nephritis for SLE. While casts were left out in the EULAR/ACR 2019 criteria, proteinuria was maintained as “>0.5 g/24 hours by 24 hours urine or equivalent spot urine protein-to-creatinine ratio”, with a relative weight of 4.

## Neurologic Disorder

Nervous system involvement is even more variable in SLE. For the ACR 1982 criteria, five manifestations were considered, namely coma, dementia, focal neurological deficit, psychosis, and seizure [5]. Two manifestations then remained for the definition of neurologic disorder, namely seizures and psychosis, both in the absence of offending drugs or known metabolic derangements, e.g., uremia, ketoacidosis, or electrolyte imbalance [5]. These two reached sensitivities of 12 and 13%, respectively, with a specificity of 99% [5]. The SLICC group in their 2012 criteria under “Neurologic” list seizures, psychosis, mononeuritis multiplex (in the absence of other known causes such as primary vasculitis), myelitis, peripheral or cranial neuropathy (in the absence of other known causes such as primary vasculitis, infection, and diabetes mellitus), and acute confusional state (in the absence of other causes, including toxic/metabolic, uremia, drugs) [7]. With the EULAR/ACR classification criteria unfolding, most of these items were again left, mostly for very low sensitivity. Seizures remained and so did psychosis, but at the same time it became apparent that much of what was originally subsumed under lupus psychosis was in fact delirium according to current neuropsychiatric concepts, characterized by (1) change in consciousness or level of arousal with reduced ability to focus, (2) symptom development over hours to < 2 days, (3) symptom fluctuation throughout the day, (4) either (4a) acute/subacute change in cognition (e.g., memory deficit or disorientation) or (4b) change in behavior, mood, or affect (e.g., restlessness, reversal of sleep/wake cycle). Psychosis is defined by delusions and/or hallucinations without insight in the absence of delirium. These three items were then also weighted differently, with relative weights of 2 for delirium, 3 for psychosis, and 5 for seizures.

## Hematologic Disorder

The various forms of cytopenia possible in SLE also underwent changes in definitions over time. The ACR 1982 criteria defined four possible manifestations within “Hematologic disorder,” namely hemolytic anemia with reticulocytosis, leukopenia defined as less than 4000/mm^3^ total on 2 or more occasions, lymphopenia defined as less than 1500/mm^3^ on 2 or more occasions or thrombocytopenia defined as less than 100,000/mm^3^ in the absence of offending drugs [5]. The SLICC 2012 criteria redefined leukopenia as < 4000/mm^3^ at least once (in the absence of other known causes such as Felty’s syndrome, drugs, and portal hypertension) and lymphopenia as < 1000/mm^3^ at least once (in the absence of other known causes such as corticosteroids, drugs, and infection) [7]. Importantly, the SLICC group broke the domain into three items, namely hemolytic anemia, leukopenia or lymphopenia, and thrombocytopenia [7]. All of these changes were re-evaluated within the EULAR/ACR classification criteria project, where, in a first step, the external SLE experts in the nominal group technique exercise removed lymphopenia. While the redefinition of leukopenia by the SLICC group stood the test, outperforming the older ACR definition, the partition of the domain did not. In fact, relevant associations were formed between cytopenias, leading to the re-introduction of the hematologic domain for the EULAR/ACR criteria. This domain now contains autoimmune hemolysis (defined as evidence of hemolysis, such as reticulocytosis, low haptoglobin, elevated indirect bilirubin or elevated LDH and a positive Coombs’ (direct antiglobulin) test) and thrombocytopenia, each with a relative weight of 4, and leukopenia with a weight of 3.

## New in EULAR/ACR Criteria: Fever

The one entirely new item for the EULAR/ACR 2019 classification criteria is fever. Fever was proposed in phase I of the project, after the project’s patient survey with the German SLE patient group had found fever reported by 54% of the patients for the time before and around their SLE diagnosis [19] and identified as a significant predictor of SLE in the project’s early SLE cohort, where 35% of the early SLE patients, but only 14% of the patients with mimicking conditions had fever [20•]. Moreover, 28% of the early SLE patients, but only 8% of the patients with mimicking diseases had fever without a relevant CRP increase [20•]. Defined as a temperature > 38.3 °C [10], fever was attributed a relative weight of 2 [8•, 9•]. Fever also played a role in the discussions on exclusion criteria. It is obvious that only non-infectious fever is meant, and there were early discussions on how to define appropriate work-up, also in light of the fact that severe bacterial infections (mostly with clearly elevated CRP levels) are a major cause of death in SLE patients. The generic definition that any item should only be attributed to SLE and thus counted if there was no more likely alternative explanation [10, 11] takes care of misclassification. However, it is important to stress this attribution rule also with regard to fever.

## Immunologic Disorder

The ACR 1982 criteria defined immunologic disorder as either positive LE cell preparation or antibody to native DNA in abnormal titer or presence of antibody to Sm nuclear antigen or a false-positive serologic test for syphilis known to be positive for at least 6 months and confirmed by *Treponema pallidum* immobilization or fluorescent treponemal antibody absorption test [5]. In the 1997 revision of the ACR criteria, the LE cell preparation was omitted, given that it was practically out of use (despite discovery of the nature of this phenomenon in the very same year) [6]. Importantly, anti-phospholipid antibodies had in between entered the stage [21], and the false-positive syphilis serology definition was changed to a “positive finding of anti-phospholipid antibodies based on (1) an abnormal serum level of IgG or IgM anticardiolipin antibodies, (2) a positive test result for lupus anticoagulant using a standard method, or (3) a false-positive serologic test for syphilis known to be positive for at least 6 months and confirmed by *Treponema pallidum* immobilization or fluorescent treponemal antibody absorption test [6]. The SLICC 2012 criteria introduced a total of 5 immunologic criteria in addition to ANA (below), namely (1) an anti-dsDNA antibody level above laboratory reference range (or ≥ 2-fold the reference range if tested by ELISA), (2) presence of an antibody to Sm nuclear antigen, (3) anti-phospholipid antibody positivity (as determined by any of a positive test result for lupus anticoagulant, a false-positive test result for rapid plasma reagin, a medium- or high-titer anticardiolipin antibody level (IgA, IgG, or IgM), or a positive test result for anti-β2-glycoprotein I (IgA, IgG, or IgM)), (4) low complement (low C3, low C4, or low CH50), and (5) a direct Coombs’ test in the absence of hemolytic anemia [7]. The association analysis within the EULAR/ACR criteria effort showed associations between antibodies to Sm and to double-stranded DNA (dsDNA) and, expectedly, between the anti-phospholipid antibodies. Therefore, the lupus-specific antibodies to Sm and dsDNA were grouped into one domain, anti-phospholipid antibodies into a second, and low complements into a third. The latter addition was also seen as an important advance in the SLICC criteria. For anti-dsDNA, there were significant concerns that tests of lesser specificity would lead to misclassification. Therefore, and in view of significant advances in the field of serology, it was decided to define anti-dsDNA by a positive result in a test that was proven to be at least 90% specific against relevant disease controls [8•, 9•]. This would typically apply to Crithidia and Farr assays. With this high level of specificity, both SLE-specific autoantibodies were attributed an equal weight of 6. In comparison, positive anti-phospholipid antibodies, defined as “anticardiolipin antibodies (IgA, IgG, or IgM) at medium or high titer (> 40 APL, GPL, or MPL, or > the 99th percentile) or positive anti-β2-glycoprotein I antibodies (IgA, IgG, or IgM) or positive lupus anticoagulant” carry only 2 points. For low complement proteins, either low C3 or low C4 have a relative weight of 3 in the EULAR/ACR criteria, while C3 and C4 both below their limits of normal are attributed 4 points [8•, 9•].

## Antinuclear Antibody

**Fig. 1:**
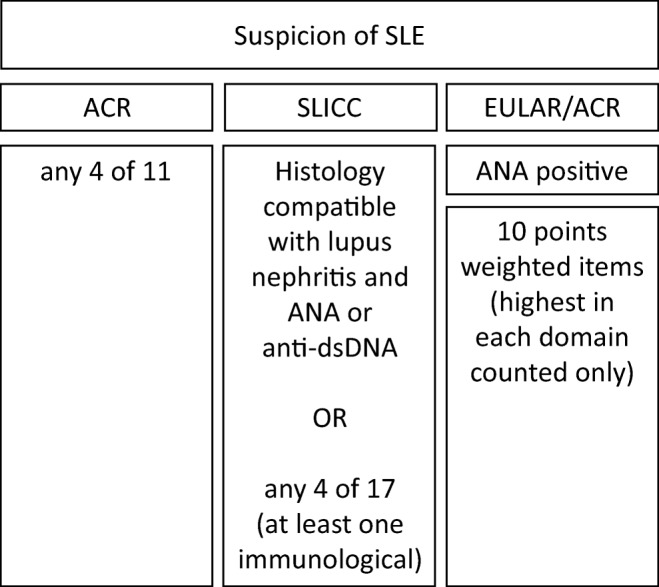
The overall structure for classification according to the ACR 1982 and 1997, the SLICC 2012, and the EULAR/ACR 2019 classification criteria for SLE

The eleventh item listed in the ACR criteria is “antinuclear antibody,” defined as an abnormal titer of antinuclear antibody by immunofluorescence or an equivalent assay at any point in time and in the absence of drugs known to be associated with “drug-induced lupus” syndrome [5]. This criterion had a sensitivity of 99%, but a specificity of only 49% [5]. Transferred to the SLICC criteria, and defined as antinuclear antibody (ANA) level above laboratory reference range, positive ANA had a sensitivity of 97% and a specificity of 45% [7]. These performance characteristics, which are well in line with the use of ANA as a screening test in clinical routine, led us to reconsider the position of ANA for the EULAR/ACR 2019 criteria. Using ANA similar to specific items seemed suboptimal. Leaving out ANA, on the other hand, would leave out a concept important for SLE. Modeling the routine situation of a screening test, we therefore evaluated ANA as an entry for classification [22]. The main concern in this regard was a relevant loss in sensitivity. However, a systematic literature search and meta-regression of published data on 13,080 SLE patients showed that at a low cut-off titer of ≥ 1:80, ANA had high sensitivity (97.8%, with a 95% confidence interval of 96.8–98.5%) [23]. Therefore, the decision was made to employ positive ANA ever as an entry criterion for the EULAR/ACR criteria [10, 24]. During the project, it became apparent that classical ANA as per indirect immunofluorescence on HEp-2 cells, were not available in all centers anymore, so that a definition of “ANA at a titer of ≥1:80 on HEp-2 cells or an equivalent positive test at least once” was adopted, highly recommending testing by immunofluorescence on HEp-2 cells or a solid-phase ANA screening immunoassay with at least equivalent performance [8, 9]. Test performance is indeed highly relevant, and given issues with ANA sensitivity of some HEp-2 and HEp-2000 test substrates [25], in particular. Structure-wise, this repositioning of ANA (Fig. 1) is one of the landmark changes towards the new EULAR/ACR criteria. This approach stood the test, with more than 99% of patients ANA positive in both the EULAR/ACR criteria derivation and validation cohorts [8, 9].

## Performance and Conclusions

The EULAR/ACR 2019 criteria have a new structure with ANA as an (obligatory) entry criterion, weighted items within domains, and a common attribution rule of counting criteria for SLE only if there is no more likely alterative diagnosis plus fever and changes to individual items. They have reached their goal of maintaining the high specificity of the ACR criteria (both 93%) while reaching a high sensitivity of 96%, not statistically different from the SLICC 2012 criteria (97%) [8, 9]. This also apparently extends to all ethnicities/races and to early disease, which is of particular importance. The new criteria now need external validation.

With regard to items, the new criteria clearly evolve from the previous sets of both the ACR 1982 [5] and 1997 [6] and the SLICC 2012 criteria [7]. It is reassuring that what we have been doing for decades is supported by significant additional evidence from current studies. The only new item is (non-infectious) fever, which should help in identifying patients with early SLE. It is important to reiterate two points that have not changed since the 1982 ACR criteria: First, classification is not the same as diagnosis [22, 26, 27]. Diagnosis is not dependent on fulfilling classification criteria, but remains with the appropriately trained physician [8•, 9•]. All attempts to withhold therapies from SLE patients not fulfilling SLE classification criteria must be countered, since this would clearly be inappropriate and dangerous. Second, classification criteria are not meant for screening. They should only be employed if there is reason to believe a patient could have SLE. With these two notes of caution, we hope that the new criteria will prove useful—and depicting the process of the derivation of the items will hopefully remove fears that they be overly complicated.

## References

[1] Rahman A, Isenberg DA (2008). Systemic lupus erythematosus. N Engl J Med.

[2] Tsokos GC (2011). Systemic lupus erythematosus. N Engl J Med.

[3] Cervera R, Khamashta MA, Font J, Sebastiani GD, Gil A, Lavilla P, Doménech I, Aydintug AO, Jedryka-Góral A, de Ramón E (1993). Systemic lupus erythematosus: clinical and immunologic patterns of disease expression in a cohort of 1,000 patients. The European working party on systemic lupus Erythematosus. Medicine (Baltimore).

[4] Bertsias GK, Pamfil C, Fanouriakis A, Boumpas DT (2013). Diagnostic criteria for systemic lupus erythematosus: has the time come?. Nat Rev Rheumatol.

[5] Tan EM, Cohen AS, Fries JF, Masi AT, McShane DJ, Rothfield NF, Schaller JG, Talal N, Winchester RJ (1982). The 1982 revised criteria for the classification of systemic lupus erythematosus. Arthritis Rheum.

[6] Hochberg MC (1997). Updating the American College of Rheumatology revised criteria for the classification of systemic lupus erythematosus. Arthritis Rheum.

[7] Petri M, Orbai AM, Alarcon GS, Gordon C, Merrill JT, Fortin PR (2012). Derivation and validation of systemic lupus international collaborating clinics classification criteria for systemic lupus erythematosus. Arthritis Rheum.

[8] Aringer M, Costenbader K, Daikh D, Brinks R, Mosca M, Ramsey-Goldman R, Smolen JS, Wofsy D, Boumpas DT, Kamen DL, Jayne D, Cervera R, Costedoat-Chalumeau N, Diamond B, Gladman DD, Hahn B, Hiepe F, Jacobsen S, Khanna D, Lerstrøm K, Massarotti E, McCune J, Ruiz-Irastorza G, Sanchez-Guerrero J, Schneider M, Urowitz M, Bertsias G, Hoyer BF, Leuchten N, Tani C, Tedeschi SK, Touma Z, Schmajuk G, Anic B, Assan F, Chan TM, Clarke AE, Crow MK, Czirják L, Doria A, Graninger W, Halda-Kiss B, Hasni S, Izmirly PM, Jung M, Kumánovics G, Mariette X, Padjen I, Pego-Reigosa JM, Romero-Diaz J, Rúa-Figueroa Fernández Í, Seror R, Stummvoll GH, Tanaka Y, Tektonidou MG, Vasconcelos C, Vital EM, Wallace DJ, Yavuz S, Meroni PL, Fritzler MJ, Naden R, Dörner T, Johnson SR (2019). 2019 European League Against Rheumatism/American College of Rheumatology classification criteria for systemic lupus erythematosus. Ann Rheum Dis.

[9] Aringer M, Costenbader K, Daikh D, Brinks R, Mosca M, Ramsey-Goldman R (2019). European League Against Rheumatism/American College of Rheumatology Classification Criteria for Systemic Lupus Erythematosus. Arthritis Rheumatol.

[10] Tedeschi SK, Johnson SR, Boumpas D, Daikh D, Dorner T, Jayne D (2018). Developing and refining new candidate criteria for systemic lupus erythematosus classification: an international collaboration. Arthritis Care Res (Hoboken).

[11] Tedeschi SK, Johnson SR, Boumpas DT, Daikh D, Dorner T, Diamond B (2019). Multicriteria decision analysis process to develop new classification criteria for systemic lupus erythematosus. Ann Rheum Dis.

[12] Tani C, D’Aniello D, Delle SA, Carli L, Cagnoni M, Possemato N (2013). Rhupus syndrome: assessment of its prevalence and its clinical and instrumental characteristics in a prospective cohort of 103 SLE patients. Autoimmun Rev.

[13] Amezcua-Guerra LM, Springall R, Marquez-Velasco R, Gomez-Garcia L, Vargas A, Bojalil R (2006). Presence of antibodies against cyclic citrullinated peptides in patients with ‘rhupus’: a cross-sectional study. Arthritis Res Ther.

[14] Aringer Martin, Costenbader Karen H., Dörner Thomas, Johnson Sindhu R. (2020). Reply. Arthritis & Rheumatology.

[15] Johnson SR, Naden RP, Fransen J, van den Hoogen F, Pope JE, Baron M, Tyndall A, Matucci-Cerinic M, Denton CP, Distler O, Gabrielli A, van Laar J, Mayes M, Steen V, Seibold JR, Clements P, Medsger TA Jr, Carreira PE, Riemekasten G, Chung L, Fessler BJ, Merkel PA, Silver R, Varga J, Allanore Y, Mueller-Ladner U, Vonk MC, Walker UA, Cappelli S, Khanna D (2014). Multicriteria decision analysis methods with 1000Minds for developing systemic sclerosis classification criteria. J Clin Epidemiol.

[16] Albrecht J, Berlin JA, Braverman IM, Callen JP, Connolly MK, Costner MI, Dutz J, Fivenson D, Franks AG, Jorizzo JL, Lee LA, McCauliffe D, Sontheimer RD, Werth VP (2004). Dermatology position paper on the revision of the 1982 ACR criteria for systemic lupus erythematosus. Lupus.

[17] Sontheimer RD (2005). Subacute cutaneous lupus erythematosus: 25-year evolution of a prototypic subset (subphenotype) of lupus erythematosus defined by characteristic cutaneous, pathological, immunological, and genetic findings. Autoimmun Rev.

[18] Touma Z, Cervera R, Brinks R, Lorenzoni V, Tani C, Hoyer BF, et al. Associations among classification criteria items within systemic lupus erythematosus. Arthritis Care Res (Hoboken). 2019. 10.1002/acr.24078. 10.1002/acr.24078 31560454

[19] Leuchten N, Milke B, Winkler-Rohlfing B, Daikh D, Dorner T, Johnson SR (2018). Early symptoms of systemic lupus erythematosus (SLE) recalled by 339 SLE patients. Lupus.

[20] Mosca M, Costenbader KH, Johnson SR, Lorenzoni V, Sebastiani GD, Hoyer BF (2019). Brief report: how do patients with newly diagnosed systemic lupus erythematosus present? A multicenter cohort of early systemic lupus erythematosus to inform the development of new classification criteria. Arthritis Rheumatol.

[21] Harris EN, Gharavi AE, Boey ML, Patel BM, Mackworth-Young CG, Loizou S, Hughes GR (1983). Anticardiolipin antibodies: detection by radioimmunoassay and association with thrombosis in systemic lupus erythematosus. Lancet.

[22] Aringer M, Dorner T, Leuchten N, Johnson SR (2016). Toward new criteria for systemic lupus erythematosus-a standpoint. Lupus.

[23] Leuchten Nicolai, Hoyer Annika, Brinks Ralph, Schoels Monika, Schneider Matthias, Smolen Josef, Johnson Sindhu R., Daikh David, Dörner Thomas, Aringer Martin, Bertsias George (2018). Performance of Antinuclear Antibodies for Classifying Systemic Lupus Erythematosus: A Systematic Literature Review and Meta-Regression of Diagnostic Data. Arthritis Care & Research.

[24] Johnson SR, Khanna D, Daikh D, Cervera R, Costedoat-Chalumeau N, Gladman DD, Hahn BH, Hiepe F, Sánchez-Guerrero J, Massarotti E, Boumpas DT, Costenbader KH, Jayne D, Dörner T, Kamen DL, Mosca M, Ramsey-Goldman R, Smolen JS, Wofsy D, Aringer M (2019). Use of consensus methodology to determine candidate items for systemic lupus erythematosus classification criteria. J Rheumatol.

[25] Pisetsky DS, Spencer DM, Lipsky PE, Rovin BH (2018). Assay variation in the detection of antinuclear antibodies in the sera of patients with established SLE. Ann Rheum Dis.

[26] Johnson SR, Goek ON, Singh-Grewal D, Vlad SC, Feldman BM, Felson DT, Hawker GA, Singh JA, Solomon DH (2007). Classification criteria in rheumatic diseases: a review of methodologic properties. Arthritis Rheum.

[27] Aringer M, Costenbader KH, Dorner T, Johnson SR. Difference between SLE classification and diagnosis and importance of attribution. Response to: ‘Do the 2019 EULAR/ACR SLE classification criteria close the door on certain groups of SLE patients?’ by Chi et al. Ann Rheum Dis. 2019. 10.1136/annrheumdis-2019-216338.

